# Objectivation of an Educational Model in Cranial Osteopathy Based on Experience

**DOI:** 10.3390/medicina57030246

**Published:** 2021-03-05

**Authors:** Jesús Requena-García, Evelyn García-Nieto, David Varillas-Delgado

**Affiliations:** 1 Formación Belga-Española de Osteopatía (FBEO), 28043 Madrid, Spain; jesus-requena@hotmail.com; 2 Higher Technical School of Industrial Engineers, Universidad Politécnica de Madrid (UPM), 28006 Madrid, Spain; evelyn.garcianieto@gmail.com; 3 Research Unit, Faculty of Medicine, Universidad Francisco de Vitoria, Pozuelo de Alarcon, 28223 Madrid, Spain

**Keywords:** osteopathic manipulation, cranial osteopathy, reproducibility, osteopathic medicine, integrative medicine

## Abstract

*Background and Objectives*: The techniques directed to the cranial field in osteopathy are the most questioned due to the lack of scientific evidence. In osteopathic practice, manual palpation is essential and, therefore, measuring reliability is fundamental. The objective of this study is to assess the precision and objectification of an educational model in cranial osteopathy based on experience. *Materials and Methods*: A reliability study was conducted in a cadaver skull where a strain gauge was placed on the sphenobasilar synchondrosis (SBS) of the base of the skull. Three cranial osteopathic techniques (lateral compression, anteroposterior compression, and compression maneuver of the mastoids) were performed 25 times by osteopaths with different degrees of experience (5–10 years, 1–5 years, <1 year). Measurements were computed for each of the three techniques of each group in comparison with the osteopath with >15 years of experience. Data were analyzed to check for inter- and intra-observer reliability using intra-class correlation coefficients (ICC). *Results*: Reliability in osteopaths with 5–10 years’ experience (observer 1 and observer 2) performing all three techniques was higher (*p* < 0.001) than the osteopath with >15 years’ experience. Little or no reliability were observed in osteopaths with less experience. *Conclusions*: The experience of the osteopaths determines the reliability and effectiveness of the cranial techniques, a fundamental part in objectifying these techniques. This model can help implement objective training in cranial osteopathy formation.

## 1. Introduction

As a discipline, osteopathy was founded in the USA in 1855 by Andrew Taylor Still [1]. Osteopathy is very effective in conditions such as the management of musculoskeletal conditions, particularly for low back pain [2,3,4], although further research is needed. Regulation of the practice of osteopathy varies in each country, sometimes depending on whether practitioners are recognized in the medical community or not [5,6]. This increasing interest in osteopathic medicine reflects society’s reality since patients turn to complementary or alternative treatments when conventional treatments fail to produce the desired result or produce side effects [7,8]. Among the available Osteopathic Manipulative Treatment (OMT) techniques, those addressed to the cranial field are the most questioned because of the lack of evidence in osteopathy effectiveness [9], efficacy [10], and mechanism of action [11]. A systematic review of 7 randomized controlled trials revealed that previous studies showed poor methodological quality and insufficient data to draw a conclusion about Osteopathy in the Cranial Field (OCF) [6]. One of the keystones of OCF teaching and practice is the presence of Primary Respiratory Mechanism (PRM), which is defined by Sutherland as the intrinsic rhythmic movement that has a structure, in this case, the skull [12]. 

According to the original model of cranial osteopathy, intrinsic rhythmic movements of [13] the human brain cause rhythmic fluctuations of cerebrospinal fluid and specific relational changes among dural membranes, cranial bones, and the sacrum [14]. Practitioners believe that they can palpably modify the parameters of this mechanism to a patient’s health advantage [14]. This rhythm represents the interaction between the tissues and fluids that make up the structure, with which it can be used as a diagnosis. Practitioners who use OCF suggest that the PRM can be palpated through the suture of the skull, however, there is a lack of evidence supporting this [13,15]. Currently, these rhythms or fluctuations have been measured through imaging tests, like physiological pulsations of the brain and the different frequency bands [16,17]. 

It has been shown that rhythm is currently measurable because concluding measurements suggest inherent motion in calvarial structures and adds to the body of evidence supporting the biomechanically measurable general calvarial motion [18]. The fact that the total intracranial area appears to expand and recede is consistent with theory and previous studies that suggest the movement of the calvarial structure due to changes in the volume of the intracranial fluid. The use of magnetic resonance technology has demonstrated the movement of the calvarial structure at a level above the threshold of resolution and provides a means for further investigation of phenomena related to the cranial concept [18]. 

It may be a matter of time before the increased resolution of MRI technology and image analysis provides the ability to examine specific movement areas of the skull bone in more detail.

Cranial osteopathy (CO) has demonstrated its efficacy in different pathologies and dysfunctions since it began with Sutherland [19] in 1940. In a recent systematic review [20], we learned that osteopathic manipulation is effective against headaches as a result of the manipulation of the cranial bones and related connective tissue.

A study by Haller et al. [21] showed that CO is effective and safe in reducing neck pain intensity, ameliorating functional disability, and improving quality of life. In another systematic review on the effects of CO [22], we learned that, although the results are heterogeneous and insufficient, the effectiveness of the CO is well documented, showing positive results in most reviewed studies, which confirms its clinical benefits. 

One of the physiological bases on which this treatment’s efficacy is based is mechanotransduction [23,24]. Still, for this effect to occur in the desired anatomical area, the mechanical load must be performed in that area.

One of the key aims of this study was to confirm whether structural changes occur in manipulating the cranium and if these can be measured. Structural changes have been demonstrated in different studies, such as that of Kostopoulos and Keramidas [25], which provides scientific evidence that forces applied to the skull through CO techniques produce an elongation of the falx cerebri. Another study demonstrating structural changes [26] concluded that cranial bone mobility can be documented and measured with X-rays. 

The available literature examining the intra- and inter-observer reliability of diagnostic procedures used in CO, like PRM-frequency, the mean duration of the flexion phase, and the mean ratio of flexion- to extension-phase and full flexion phase of the cranial rhythmic impulse (CRI) reports consistent findings with methodological quality [13,27,28]. 

To validate the manipulation of the OCF, it is necessary to demonstrate when performing the techniques that there is a mechanical force applied to specific cranial anatomical structures. Through this, mechanical energy changes are produced in the tissues. To measure whether mechanical loads occur, we used strain gauges. These instruments have already been used in several academic studies [29].

Because the maneuvers are precise, we want to observe and measure if the experience is relevant in this procedure. The more a gesture is repeated, the better and more precise it will be executed [24]. This study’s objective was to evaluate the precision of osteopathic manipulation of the skull according to the degree of experience and assess it as an educational model for students’ training.

## 2. Materials and Methods

### 2.1. Participants

Professional osteopaths of *Formación Belga-Española de Osteopatía* (FBEO) were selected based on their degree of experience. An osteopath with more than 15 years of experience in cranial osteopathy was selected and classified as the most experienced measurement professional. Two osteopaths (observer 1 and observer 2) with 5–10 years of experience, 2 osteopaths with 1–5 years of experience, and 2 osteopaths with less than 1 year of experience were selected to carry out the techniques and assess inter-observer and intra-observer reliability. Osteopaths trained in FBEO were classified based on their degree of experience in the use of the OCL field and selected to participate in our study. All the selected osteopaths agreed voluntarily to take part in the study. The age range of the participants was 23–43 years old. Before enrolment in the study, all osteopaths were fully informed of the experimental procedures and signed informed written consent. The study was approved by the Francisco de Vitoria University Research Ethics Committee (33-2019) and was conducted in accordance with the Declaration of Helsinki (last modification in 2013).

### 2.2. Material of Assays

An open cadaver skull was used to perform measurements in a structure that was most like the daily consultation. For the study, an adult’s skull was used, the same one for all measurements. 

### 2.3. Measurements 

**Figure 1:**
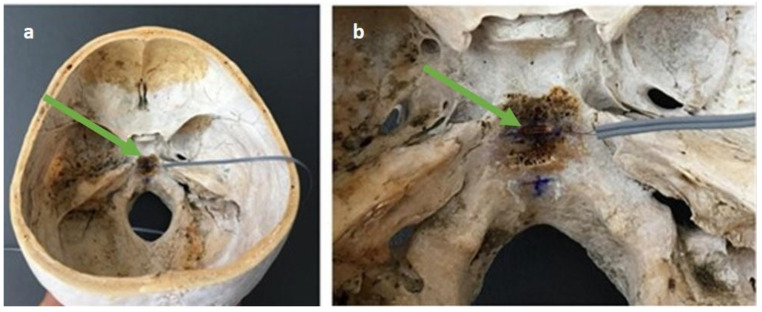
(**a**) Skull base and the arrangement of gauge. (**b**) Gauge placed at the sphenobasilar synchondrosis (SBS) of the base of the skull.

A strain gauge with a rectangular shape was placed on the SBS following the anatomical structure of this joint, as shown in Figure 1. The gauge was attached to the skull’s bony substance using electrodes connected to the computer. The software used to obtain the readings was described below.

**Figure 2:**
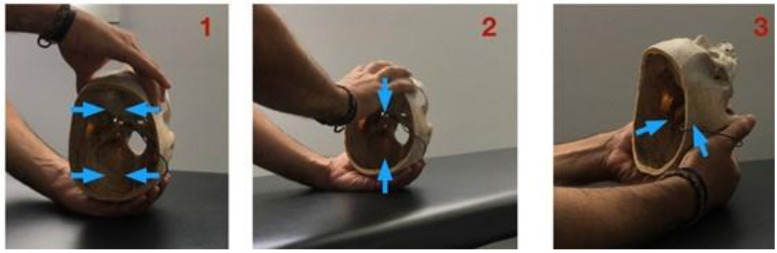
The different maneuvers were carried out for the evaluation of the study. (**1**) Lateral compression manoeuvre, (**2**) anteroposterior compression manoeuvre, and (**3**) compression manoeuvres of the mastoids.

Three cranial osteopathy (CO) techniques were repeated 25 times each by osteopaths bringing the maximum load to that structure and trying to apply the same load each time. In this way, the accuracy of the osteopath could be determined. The 3 techniques selected are shown in Figure 2. Those were:

1. *Lateral compression* (technique 1): The osteopath places one hand on the occipital and the other on the main wings of the sphenoid. The anterior hand makes an internal pressure through the major wings of the sphenoid, the posterior hand makes an internal pressure through the occipital squama.
2. *Anteroposterior compression* (technique 2): Osteopath’s hands are placed on the occipital and on the frontal bone. The maneuver consists of performing an integer-posterior pressure producing compression of the SBS.
3. *Compression of mastoids* (technique 3): The osteopath places both thumbs and thenar eminences on the mastoid processes. The action consists of pressing both mastoids in the direction of the SBS.

All the information about the technique-induced deformations was registered using the MM01_multidaq software (Micro-Measurements, Vishay Precision Group, Inc. (VPG), 3 Great Valley Parkway, Malvern, PA, USA, E.E.U.U.), providing values in micrometers (μm). Values indicate the micrometers of deformation. Positive values indicate stretching, whereas negative values indicate compression. 

To minimize possible biases in the measurements, the selected osteopaths performed 25 repetitions of each technique.

### 2.4. Statistical Analysis 

The results of each test were blindly introduced into the statistical package SPSS v 20.0 (IBM SPSS Statistics, IBM Corporation, Armonk, NY, USA) and analyzed afterward. The mean and standard deviation (SD) were used for descriptive purposes. 

For the measurements, the intra- and inter-rater reliability, based on consistency, were analyzed by determining the two-way mixed-effects ICC (ICC (2,1)) values and 95% confidence intervals (CIs). The ICC (2,1) was interpreted according to the classification system: 0–0.25 indicating “*little or no reliability*”; 0.26–0.49 indicating “*low reliability*”; 0.50–0.69 indicating “*moderate reliability*”; 0.70–0.89 indicating “*high reliability*”; and 0.90–1.00 indicating “*very high reliability*.” 

In all statistical tests, a level of *p* < 0.05 was set to establish statistically significant differences.

## 3. Results

Data of measurements made in the three techniques by osteopaths are shown in Table 1.

Reliability between osteopath with >15 years of experience and osteopaths with 5–10 years of experience was high for all three techniques with ICC values 0.809, 0.851, and 0.861 (95%CI: 0.444 to 0.924, 0.620 to 0.938, and 0.678 to 0.939, respectively) for observer 1 (ICC: *p* <0.001), similar to observer 2 (Table 2). In the first technique, little or no reliability was observed in osteopaths with 1–5 years of experience. The results for observer 1 were 0.015 (95CI%: −0.051 to 0.067) (*p* = 0.783) and the results for observer 2 were 0.036 (95%CI: −0.042 to 0.184) (*p* = 0.683), with similar results in technique 2 (Table 2). However, in technique 3, in osteopath with 1–5 years of experience, low reliability was found in observer 2 with ICC value 0.390 (CI95%: −0.127 to 0.766) (*p* = 0.152) and high reliability in observer 1; 0.806 (95%CI: 0.565 to 0.914) (*p* <0.001). The observers with less than one year of experience presented little or no reliability in technique 1 and technique 2, and a low concordance in technique number 3 concerning an osteopath with >15 years of experience (observer 1 0.416 (95%CI: −0.209 to 0.769); *p* = 0.392 and observer 2 0.397 (95%CI: −0.124 to 0.772); *p* = 0.203) (Table 2).

Measurements of three CO techniques were made among the observers with different degrees of experience to verify the average effect of the observers per group concerning the osteopath with >15 years of experience. The reliability in the average to osteopaths with 5–10 years of experience was high by the ICC in technique 1 (ICC; 0.760 (95%CI: 0.288 to 0.906) (*p* < 0.001)), technique 2 (ICC; 0.856 (95%CI: 0.499 to 0.946) (*p* < 0.001)) and technique 3 (ICC; 0.856 (95%CI: 0.627 to 0.940) (*p* < 0.001)). Little or no reliability was observed in the averages to observers with less experience. However, in technique 3, low reliability was found in osteopaths with 1–5 years of experience and less than one year of experience (Table 3).

## 4. Discussion

Although the efficacy of CO has been demonstrated in several fields, as far as we know, this is the first study on reliability and objectification learning in the field of CO. Further research on action mechanisms and precision in manipulations is required to provide osteopathy professionals with higher quality preparation.

In addition to demonstrating structural changes, further studies have been carried out on the mechanical load needed to produce such changes. The study by Downey et al. [30] simulated a craniosacral treatment technique: Frontal lift by applying accurately measured distractive forces and a change in intracranial pressure and movement through the coronal suture were observed in an animal with forces greater than those used clinically in the practice of CO (traction of 1000 gr), according to the results and clinical implications presented in this work. Another study in the same line of investigation is the review about the cranial motion by Seimetz et al. [31], where it is illustrated that both externally applied forces and increased intracranial pressure result in measurable movement through cranial sutures in young and adult mammals and measurable changes in the diameter of the cranial vault in human skulls of living and post-mortem adults. However, the magnitude of cranial movement may vary depending on the subject, the head region where forces are applied, and the force application method. This last conclusion of the study by Seimetz et al. [31] highlights the importance of the different methods to apply forces in the skull, which agree with our study.

The great pending conversation in CO, which also motivates this study, is diagnostic evaluation. There are studies that show differences in tactile ability, such as the study by Nascimiento et al. [32], which states that teaching strategies used during different educational periods can contribute to improving tactile sensitivity and precision through professionals in manual palpation. In this respect, another study investigated the effects of standardized protocol training on cranial palpation pressures used by osteopathy professionals [33], and concluded that palpatory training was ineffective in improving accuracy in professionals of cranial palpation, also suggesting to examine palpation pressures used by experienced professionals. Both studies [32,33] ultimately highlighted the importance of training in professional cranial palpation, which is in agreement with our results.

A study assessing the inter and intra-observer reliability in palpation of the primary respiratory mechanism within the cranial concept was carried out by Sommerfeld et al. [13]. Their results showed that PRM could not be reliably palpated and, under certain conditions, was influenced by the respiratory rates of examiners. These results do not support the hypotheses based on the role of PRM palpation for clinical decision making.

Currently, the cranium can be considered a model of tensegrity and functional unity as stated in the study of Scarr et al. [34], where it was concluded that tension forces in dura mater have the effect of separating bones as well as integrating them into a single functional unit. This alteration of the dura mater or suture occurs in a specific anatomical area of the cranium. Therefore, it is important for the reliability of both diagnosis and treatment that osteopaths know with precision which anatomical structure they are palpating.

If the treatment approach is assessed, mechanotransduction [35] could be the scientific explanation for it, understanding it as the mechanisms by which cells convert mechanical stimuli into cellular responses, what underlines the importance of precision in the treatment, making sure to exert the correct mechanical load on the right place. The data obtained in this study confirm the importance of precision when performing CO techniques.

Another issue to be solved was whether hands could perceive movements and minimal changes that occur in the cranial tissue. In this regard, the study by Kasparian et al. [35] determined that one-third of our samples were capable to detect movements of less than 50 μm. 

For this reason, the authors of this article agreed that the cranium should not be treated like any other body structure [36].

Our study can contribute to scientific progress in this area, making a precision model of reliability, evaluation, and teaching strategies. As described before, CO has a lot of beneficial effects on human health with a scientific and physiological basis in the principles of mechanotransduction. To date, there is no model of cranial manipulation that teaches with precision how to perform techniques or mechanical loads to act on specific anatomical structures of the cranium, which can translate into measurable clinical outcomes. As shown in the data of this study, there are significant differences between osteopaths based on the degrees of experience, and this model could be used to level any student and bring them to a quantifiable standard.

The data obtained in this study show that cranial osteopathic treatment depends not only on the osteopath’s subjective tactile sensitivity but that it is an area of manual manipulative treatment that can be trained and practiced in an objective manner to achieve measurable results.

There are differences between readings of osteopaths with different degrees of experience, which shows that experience is fundamental when it comes to possible clinical outcomes.

The limitations of this study were; all osteopaths in the study come from the same school (Formación Belga-Española de Osteopatía), thus the results cannot be extrapolated and represent the entire osteopathic community in our country. The school is accredited by the Federation of Osteopaths of Spain (FOE), showing reliable data in the field of research due to specialized training. The osteopathic approach to an in vivo head is different from a skull cadaver and even more from a skull base cadaver, as in this study. However, these results provide knowledge about the methodology and offer an area of improvement in teaching methodology for cranial osteopathy. Using a biomechanical tool in which clear and objective feedback is obtained on how forces are directed through the skull would benefit osteopathy training.

The data presented opens a new possibility in the teaching and training of osteopaths through the objectification of the manipulations, measuring the reliability of the techniques, thus that the operator knows in which anatomical structure he is directing his load. In this way, he can be accurate in performing the cranial osteopathy treatment techniques used for the patient’s rehabilitation.

## 5. Conclusions

This study supports the capacity of precision that an osteopath has in exerting a mechanical load on a specific anatomical structure. The mechanical load was applied to a cadaver skull base in our study. This gave reliability and security to the treatment carried out in the clinical setting. The ability of the examiner to perform this action on the SBS in a precise and repetitive manner is determined. Therefore, we conclude and confirm greater reliability and effectiveness in cranial osteopathic techniques in professionals with more years of experience compared to those with fewer years.

As these results can be measured, it is concluded that the precision in the direction that the therapist must perform in cranial maneuvers can be trained, improved, and objectified.

## Figures and Tables

**Table 1:** Measurement data in micrometers (µm) and standard deviation (SD) of osteopaths in the three techniques.

		Technique 1		Technique 2		Technique 3	
		Mean (SD)	[95%CI]	Mean (SD)	[95%CI]	Mean (SD)	[95%CI]
**>15 years**		−63.92 (12.721)	[−69.17 to −58.67]	44.48 (7.880)	[41.43 to 47.73]	−56.28 (10.872)	[−60.77 to −51.79]
**5–10 years**	*Observer 1*	−56.68 (17.509)	[−63.91 to −49.45]	41.16 (10.896)	[36.66 to 45.66]	−53.36 (11.090)	[−57.94 to −48.78]
	*Observer 2*	−53.92 (18.307)	[−61.48 to −46.36]	40.00 (8.869)	[36.34 to 43.66]	−51.48 (10.621)	[−56.22 to −47.46]
**1–5 years**	*Observer 1*	−6.88 (2.068)	[−7.73 to −6.03]	20.06 (3.697)	[19.07 to 22.13]	−59.08 (18.632)	[−66.77 to −51.39]
	*Observer 2*	−19.56 (3.731)	[−21.10 to −18.02]	22.92 (2.886)	[21.73 to 24.11]	−37.28 (8.142)	[−40.67 to −33.92]
**<1 year**	*Observer 1*	−6.80 (3.488)	[−8.24 to −5.36]	16.16 (4.972)	[14.11 to 18.21]	−39.40 (9.954)	[−43.51 to −35.29]
	*Observer 2*	−10.08 (4.890)	[−12.10 to −8.06]	26.72 (6.202)	[24.16 to 29.28]	−36.28 (9.374)	[−40.15 to −32.41]

**Table 2:** Reliability analysis and concordance between osteopaths with >15 years of experience and different observers based on the experience for the action of all maneuvers and standard deviation (SD) of osteopaths in the three techniques. ICC: Intraclass Correlation Coefficient.

		Technique 1				
		*Measures*	*Mean Differences (SD)*	*95% Limits of Agreement*	*ICC*	*[95%CI]*
**5–10 years**	*Observer 1—>15 years*	25	−7.240 (10.868)	−11.726 to −2.754	0.809	[0.444 to 0.924]
	*Observer 2—>15 years*	25	−10.000 (13.491)	−15.569 to −4.431	0.696	[0.173 to 0.877]
**1–5 years**	*Observer 1—>15 years*	25	−57.040 (13.843)	−62.754 to −51.326	0.015	[−0.051 to 0.067]
	*Observer 2—>15 years*	25	−44.360 (11.676)	−49.180 to −39.540	0.036	[−0.042 to 0.184]
**<1 year**	*Observer 1—>15 years*	25	−57.120 (10.635)	−61.510 to −52.730	0.035	[−0.027 to 0.170]
	*Observer 2—>15 years*	25	−53.840 (10.656))	−58.239 to −49.441	0.046	[−0.031 to 0.213]
		**Technique 2**				
		***Measures***	***Mean Differences (SD)***	***95% Limits of Agreement***	***ICC***	***[95%CI]***
**5–10 years**	*Observer 1—>15 years*	25	3.320 (6.310)	0.716 to 5.924	0.851	[0.620 to 0.938]
	*Observer 2—>15 years*	25	4.480 (6.404)	1.837 to 7.123	0.770	[0.339 to 0.909]
**1–5 years**	*Observer 1—>15 years*	25	23.880 (7.965)	20.592 to 27.168	0.038	[−0.059 to 0.205]
	*Observer 2—>15 years*	25	21.560 (7.969)	18.271 to 24.849	0.026	[−0.066 to 0.184]
**<1 year**	*Observer 1—>15 years*	25	28.320 (9.371)	24.452 to 32.188	0.002	[−0.061 to 0.109]
	*Observer 2—>15 years*	25	17.760 (7.102)	14.828 to 20.692	0.216	[−0.124 to 0.586]
		**Technique 3**				
		***Measures***	***Mean Differences (SD)***	***95% Limits of Agreement***	***ICC***	***[95%CI]***
**5–10 years**	*Observer 1—>15 years*	25	−2.920 (7.354)	−5.955 to 0.115	0.861	[0.678 to 0.939]
	*Observer 2—>15 years*	25	−4.440 (7.534)	−7.550 to −1.330	0.824	[0.529 to 0.928]
**1–5 years**	*Observer 1—>15 years*	25	2.800 (12.261)	−2.261 to 7.861	0.806	[0.565 to 0.914]
	*Observer 2—>15 years*	25	−19.000 (7.269)	−22.000 to −16.000	0.390	[−0.127 to 0.766]
**<1 year**	*Observer 1—>15 years*	25	−16.880 (9.293)	−20.716 to −13.044	0.416	[−0.209 to 0.769]
	*Observer 2—>15 years*	25	−20.000 (7.522)	−23.105 to −16.895	0.397	[−0.124 to 0.772]

**Table 3:** Reliability analysis and concordance in the means of the observations in the three techniques.

		Technique 1		Technique 2		Technique 3	
		*ICC*	*[95%CI]*	*ICC*	*[95%CI]*	*ICC*	*[95%CI]*
**5–10 years**	*Observers—>15 years*	0.760	[0.288 to 0.906]	0.856	[0.499 to 0.946]	0.856	[0.627 to 0.940]
**1–5 years**	*Observers—>15 years*	0.003	[−0.032 to 0.075]	0.032	[−0.058 to 0.191]	0.480	[−0.086 to 0.762]
**<1 year**	*Observers—>15 years*	0.040	[−0.028 to 0.191]	0.077	[−0.072 to 0.318]	0.413	[−0.142 to 0.781]

## Data Availability

The data presented in this study are available on request from the corresponding author. The data are not publicly available due to legal restrictions.

## References

[1] Hamonet C. (2003). Andrew Taylor Still and the birth of osteopathy (Baldwin, Kansas, USA, 1855). Jt. Bone Spine.

[2] Jonas C. (2018). Musculoskeletal Therapies: Osteopathic Manipulative Treatment. FP Essent..

[3] (2016). American Osteopathic Association Guidelines for Osteopathic Manipulative Treatment (OMT) for Patients With Low Back Pain. J. Am. Osteopath. Assoc..

[4] Licciardone J.C., Brimhall A.K., King L.N. (2005). Osteopathic manipulative treatment for low back pain: A systematic review and meta-analysis of randomized controlled trials. BMC Musculoskelet. Disord..

[5] Luciani E., Consorti G., van Dun P.L.S., Merdy O., Lunghi C., Petracca M., Esteves J.E., Cerritelli F. (2018). An overview of osteopathy graduates’ perceived preparedness at transition from educational environment to clinic environment one year after graduation: A cross sectional study. BMC Med. Educ..

[6] Guillaud A., Darbois N., Monvoisin R., Pinsault N. (2016). Reliability of Diagnosis and Clinical Efficacy of Cranial Osteopathy: A Systematic Review. PLoS ONE.

[7] Dossett M.L., Cohen E.M., Cohen J. (2017). Integrative Medicine for Gastrointestinal Disease. Prim. Care.

[8] Martins W.R., Diniz L.R., Blasczyk J.C., Lagoa K.F., Thomaz S., Rodrigues M.E., de Oliveira R.J., Bonini-Rocha A.C. (2015). Immediate changes in electroencephalography activity in individuals with nonspecific chronic low back pain after cranial osteopathic manipulative treatment: Study protocol of a randomized, controlled crossover trial. BMC Complement Altern. Med..

[9] Hall H., Cramer H., Sundberg T., Ward L., Adams J., Moore C., Sibbritt D., Lauche R. (2016). The effectiveness of complementary manual therapies for pregnancy-related back and pelvic pain: A systematic review with meta-analysis. Medicine.

[10] Clark B.C., Thomas J.S., Walkowski S.A., Howell J.N. (2012). The biology of manual therapies. J. Am. Osteopath. Assoc..

[11] Hastings V., McCallister A.M., Curtis S.A., Valant R.J., Yao S. (2016). Efficacy of Osteopathic Manipulative Treatment for Management of Postpartum Pain. J. Am. Osteopath. Assoc..

[12] Sutherland W.G. (2000). The cranial bowl. 1944. J. Am. Osteopath. Assoc..

[13] Sommerfeld P., Kaider A., Klein P. (2004). Inter- and intraexaminer reliability in palpation of the “primary respiratory mechanism” within the “cranial concept”. Man. Ther..

[14] Hartman S.E. (2006). Cranial osteopathy: Its fate seems clear. Chiropr. Osteopat..

[15] Sabini R.C., Elkowitz D.E. (2006). Significance of differences in patency among cranial sutures. J. Am. Osteopath. Assoc..

[16] Kiviniemi V., Wang X., Korhonen V., Keinänen T., Tuovinen T., Autio J., LeVan P., Keilholz S., Zang Y.F., Hennig J. (2016). Ultra-fast magnetic resonance encephalography of physiological brain activity—Glymphatic pulsation mechanisms?. J. Cereb. Blood Flow Metab..

[17] Strik C., Klose U., Erb M., Strik H., Grodd W. (2002). Intracranial oscillations of cerebrospinal fluid and blood flows: Analysis with magnetic resonance imaging. J. Magn. Reson. Imaging.

[18] Crow W.T., King H.H., Patterson R.M., Giuliano V. (2009). Assessment of calvarial structure motion by MRI. Osteopath. Med. Prim. Care.

[19] Bordoni B., Zanier E. (2015). Sutherland’s legacy in the new millennium: The osteopathic cranial model and modern osteopathy. Adv. Mind Body Med..

[20] Whalen J., Yao S., Leder A. (2018). A Short Review of the Treatment of Headaches Using Osteopathic Manipulative Treatment. Curr. Pain Headache Rep..

[21] Haller H., Lauche R., Cramer H., Rampp T., Saha F.J., Ostermann T., Dobos G. (2016). Craniosacral Therapy for the Treatment of Chronic Neck Pain: A Randomized Sham-controlled Trial. Clin. J. Pain.

[22] Jakel A., von Hauenschild P. (2011). Therapeutic effects of cranial osteopathic manipulative medicine: A systematic review. J. Am. Osteopath. Assoc..

[23] Ingber D.E. (1997). Tensegrity: The architectural basis of cellular mechanotransduction. Annu. Rev. Physiol..

[24] Langevin H.M., Storch K.N., Snapp R.R., Bouffard N.A., Badger G.J., Howe A.K., Taatjes D.J. (2010). Tissue stretch induces nuclear remodeling in connective tissue fibroblasts. Histochem. Cell Biol..

[25] Kostopoulos D.C., Keramidas G. (1992). Changes in elongation of falx cerebri during craniosacral therapy techniques applied on the skull of an embalmed cadaver. Cranio.

[26] Oleski S.L., Smith G.H., Crow W.T. (2002). Radiographic evidence of cranial bone mobility. Cranio.

[27] Green C., Martin C.W., Bassett K., Kazanjian A. (1999). A systematic review of craniosacral therapy: Biological plausibility, assessment reliability and clinical effectiveness. Complement Ther. Med..

[28] Moran R.W., Gibbons P. (2001). Intraexaminer and interexaminer reliability for palpation of the cranial rhythmic impulse at the head and sacrum. J. Manip. Physiol. Ther..

[29] Fechoz F., Delecrin J., Passuti N., Royer J. (1993). Mechanical behavior of the human acetabulum. Study by electric extensiometry before and after implantations of prosthetic units. Chirurgie.

[30] Downey P.A., Barbano T., Kapur-Wadhwa R., Sciote J.J., Siegel M.I., Mooney M.P. (2006). Craniosacral therapy: The effects of cranial manipulation on intracranial pressure and cranial bone movement. J. Orthop. Sports Phys. Ther..

[31] Seimetz C.N., Kemper A.R., Duma S.M. (2012). An investigation of cranial motion through a review of biomechanically based skull deformation literature. Int. J. Osteopath. Med..

[32] Nascimento L.P., Oliva-Pascual-Vaca A., Renan-Ordine R., Riquelme I., Ricard F., Rodriguez-Blanco C. (2016). Comparative assessment of tactile sensitivity between undergraduate and postgraduate health sciences students. Int. J. Osteopath. Med..

[33] Zegarra-Parodi R., de Chauvigny de Blot P., Rickards L.D., Renard E.O. (2009). Cranial palpation pressures used by osteopathy students: Effects of standardized protocol training. J. Am. Osteopath. Assoc..

[34] Scarr G. (2008). A model of the cranial vault as a tensegrity structure, and its significance to normal and abnormal cranial development. Int. J. Osteopath. Med..

[35] Kasparian H., Signoret G., Kasparian J. (2015). Quantification of Motion Palpation. J. Am. Osteopath. Assoc..

[36] Basic-Kes V., Basic-Jukic N., Kes P., Demarin V., Labar B. (2002). Neurologic sequelae of bone changes in multiple myeloma and its therapy. Acta Med. Croat..

